# Closure of open window thoracostomy due to MRSA pyothorax by EWS bronchial occlusion and modified extraperiosteal air plombage: a case report

**DOI:** 10.1186/s40792-024-01815-y

**Published:** 2024-01-17

**Authors:** Masami Kuramochi, Mayumi Shinonaga, Setsuo Kuraoka

**Affiliations:** Department of Thoracic Surgery, Mito Saiseikai General Hospital, 3-3-10 Futabadai Mito, Ibaraki, 311-4198 Japan

**Keywords:** Open window thoracostomy, Fenestration, Pyothorax, Empyema, Extraperiosteal air plombage, Endobronchial Watanabe Spigot (EWS)

## Abstract

**Background:**

Refractory pyothorax caused by methicillin-resistant *Staphylococcus aureus* (MRSA) is a challenging clinical condition; complications such as bronchopleural fistulae can further hinder its treatment. To avoid a fatal state caused by aspirating pneumonia, open window thoracotomy is not only sometimes performed, but subsequent closure of the window can also be difficult. In this report, we describe the case of a patient with MRSA pyothorax with bronchopleural fistula in whom a successful closure of window thoracostomy was achieved by utilizing Endobronchial Watanabe Spigot　(EWS; Novatech, La Ciotat, France) bronchial occlusion and a modified extraperiosteal air plombage technique.

**Case presentation:**

A 66-year-old man underwent an open window thoracotomy for pyothorax with bronchopleural fistula with MRSA infection at the age of 59. After 7 years, he was referred to our department for the closure of the window. Initially, we occluded the right B6a + b by EWS under bronchoscopy. Subsequently, we dissected the intercostal muscles between the 3rd, 4th, 5th, and 6th ribs to collapse the pyothorax cavity and ensure the coverage of the fistula of lung including the hypertrophied parietal pleura and soft tissues of the chest wall. We filled the extrapleosteal space with a pedicled anterior serratus muscle flap to compress the parietal pleura. Postoperatively, lung expansion was satisfactory, and there has been no recurrence for 6 years since the window closure surgery.

**Conclusions:**

We were able to achieve closure and healing in a patient who underwent open window thoracostomy for MRSA bronchopleural fistula by applying EWS and modified extraperiosteal air plombage technique.

## Background

Refractory methicillin-resistant *Staphylococcus aureus* (MRSA) pyothorax with bronchopleural fistula is a common challenging condition. Open window thoracostomy is usually performed for the purpose of avoiding life-threatening complications such as aspiration pneumonia caused by fistula; however, patients face significant challenges such as frequent gauze changes, bathing restrictions, and cosmetic issues. Furthermore, the timing and method of closure vary depending on factors such as comorbidities, nutritional status, infection status, and availability of tissues for filling procedures, which further complicate the decision-making process. Therefore, it is important to disseminate knowledge on closure techniques, which can accommodate various cases. In this report, we present a case of a patient who developed MRSA bronchopleural fistula after pneumothorax surgery and achieved favorable results by applying Endobronchial Watanabe Spigot (EWS) bronchial occlusion and modified extraperiosteal air plombage.

## Case presentation

**Fig. 1:**
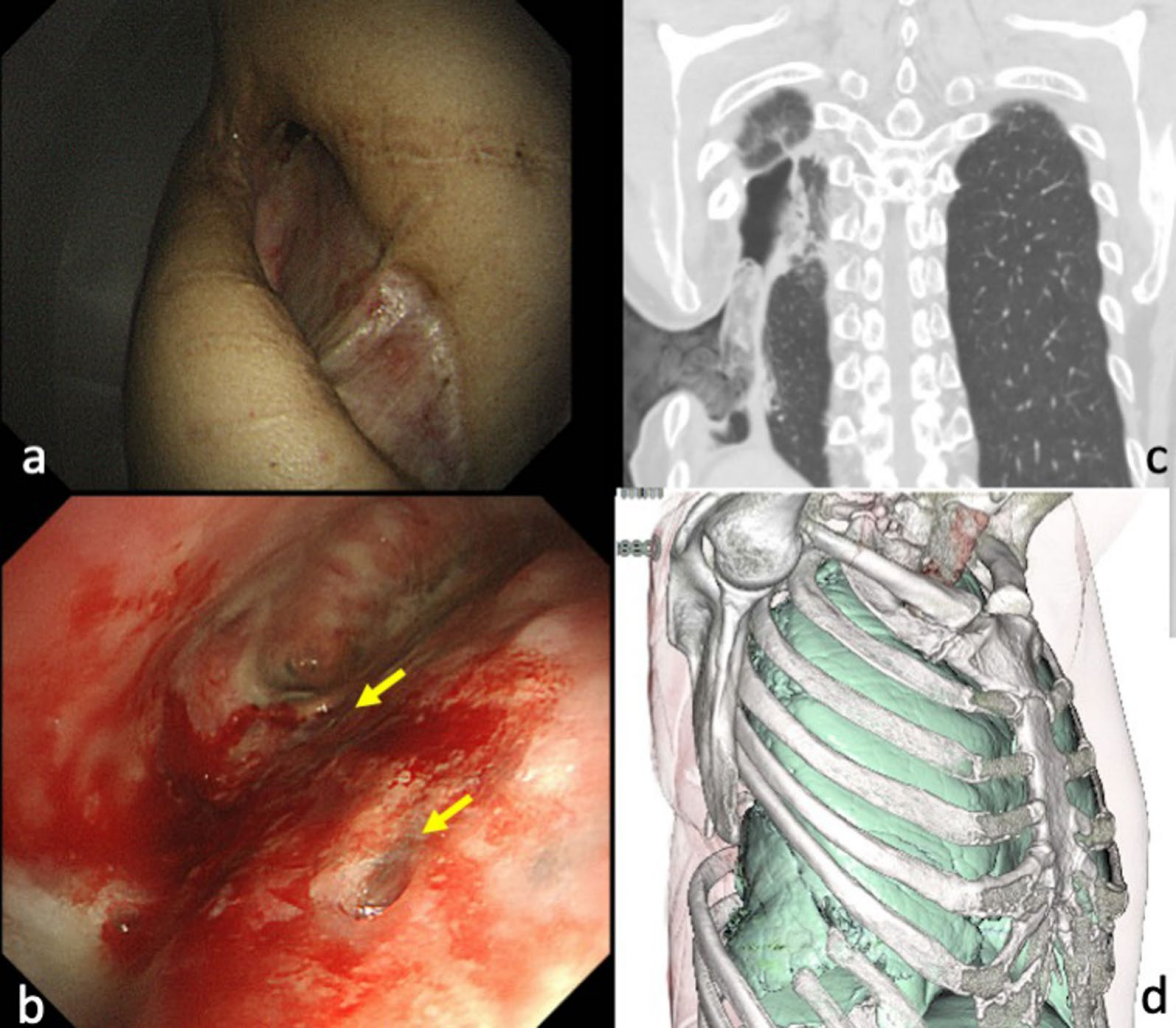
Aspect of open window on the right side of the thorax because of pyothorax. **a** At least two fistulae were observed (arrows) (**b**). CT revealed resection of 7th and 8th ribs for open window thoracotomy and cranial extension of the pyothorax cavity (**c**, **d**)

A 66-year-old man underwent right spontaneous pneumothorax surgery (thoracotomy) at the age of 23. However, he experienced a recurrence at the age of 59, which led to lung plication and partial lung resection surgery through thoracotomy once again. Two months later, he developed an MRSA pyothorax with fistulae and subsequently underwent an open window thoracostomy procedure. Following this, he maintained regular visits to the outpatient clinic for dressing changes at the window site until he was referred to our department at the age of 66 for closure. The patient had a medical history of Sweet’s syndrome, appendicitis, and gastric ulcer surgery. He had a height of 158 cm, weighed 49.4 kg, and had a slender build. There was an approximately 5 × 15 cm window opening caudal to the right subscapular angle of the chest. The pyothorax cavity extended toward the cranial side, and at least two fistulae were observed (Fig. 1a, b). Culture of the fistula discharge revealed MRSA, but there were no signs of infection such as fever. Laboratory tests showed a white blood cell count (WBC) of 6000/μl and C-reactive protein (CRP) level of 0.13 mg/dl, indicating no evidence of infection. Chest computed tomography (CT) confirmed the resection of the 7th and 8th ribs on the right side, with the pyothorax cavity spreading toward the cranial side (Fig. 1c, d). Initially, we performed bronchial occlusion using EWS to achieve closure or reduction of the bronchopleural fistula. By spraying 0.1% gentian-violet solution around the fistula through the open window, we observed the retention of the dye deep to the right B6a + b through a bronchoscope. One EWS was filled into this bronchus, while another was placed at the entrance of the right B6. Subsequently, the patient underwent a closure of the window under general anesthesia. A posterolateral incision was made from the limbus of the right open window to expose the right 2nd to 6th ribs. Then, we dissected the anterior serratus muscle from its attachment site on the humerus to create a pedicled anterior serratus muscle flap, while preserving the branches of the right thoracodorsal artery. The pyothorax cavity was thoroughly debrided and washed with more than 3 L of saline following a 0.1% gentian-violet solution. Since no air leakage was observed from the fistula during surgery, the fistula site was not sutured. The intercostal muscles between the 3rd, 4th, 5th, and 6th ribs, including the hypertrophied parietal pleura, were detached from the chest wall and compressed to collapse the pyothorax cavity, ensuring that the lung fistula was covered. Fibrin glue was applied between the detached chest wall soft tissues including parietal pleura and the lung to promote adhesion. The 4th rib was partially resected by approximately 10 cm, and a pedicled anterior serratus muscle flap was introduced through the gap, compressing the detached chest wall soft tissues including the parietal pleura to ensure their adherence to the fistula site. We utilized a monofilament absorbable suture to secure the serratus anterior to the cranial top of the plombage space. The suturing was performed at multiple points to ensure stable positioning.　On the caudal side of the open window, only the thickened parietal pleura was dissected as much as possible to create an extrapleural space, and the dissected parietal pleura was sutured to chest wall soft tissues including the parietal pleura at cranial side and closed pyothorax cavity. Finally, a drain was placed in the pyothorax cavity without passing through the plombage space, and the skin was sutured to complete the window closure. The surgery lasted for 4 h and 33 min, with a blood loss of 460 ml. The drain was removed on the fourth day postoperatively. Preoperatively, cultures of the fistula discharge revealed the presence of MRSA and Corynebacterium. Accordingly, vancomycin, meropenem, and minocycline, to which these microorganisms were sensitive, were administered for 1 week postoperatively. Subsequently, a combination of minocycline, daptomycin with good muscle and tissue penetration, and the lung-penetrating linezolid were used for 2 weeks. The patient was discharged on the 35th postoperative day.

The EWS used for the right B6 bronchial filling was spontaneously exsanguinated soon after surgery. The other EWS in the right B6a + b remained in place for a long time without any adverse events. The patient displayed a mild winged scapula; however, this did not lead to any significant limitations in activities of daily living, nor did it cause notable restrictions in shoulder elevation or induce pain.

A CT scan 6 years after surgery showed that lung expansion was maintained.

## Discussion

The treatment of chronic pyothorax primarily aims to achieve either a re-expansion of the collapsed lung, a reduction of the empyema cavity, or both, leading to the elimination of potential infected dead spaces. Various surgical techniques are available for pyothorax cavity reduction, including thoracoplasty, muscle flap interposition, omentopexy, and extrapleosteal air plombage. In this case, thoracoplasty was not considered due to the advanced scapular depression and chest wall deformity observed in the patient. Similarly, omentopexy was challenging due to the patient's history of gastric ulcer surgery and obtaining an adequate amount of muscle for flap interposition was not possible because the latissimus dorsi muscle had been previously severed. Moreover, extraperiosteal air plombage would involve a removal of the chest wall soft tissue, including the intercostal muscles, and dropping it into the pyothorax cavity, thereby reducing its size. This method creates an extrapleosteal space where the accumulated exudate can be gradually absorbed over time, facilitating re-expansion of the collapsed lung, and improving lung function. It also preserves the bony thorax, avoiding chest wall deformities [1, 2]. However, if the extrapleosteal space becomes infected, it can lead to the development of new empyema. Therefore, we decided to modify the extraperiosteal air plombage technique to control the risk of infection. Although MRSA was detected in the secretion from the fistula, it had been more than 6 years since the open window thoracostomy, and the pyothorax cavity was in a stable, non-infectious state. Therefore, we first performed an endobronchial occlusion with EWS to eliminate the lung fistula and reduce the risk of infection by the fistula secretion [3]. During the surgery, we extensively covered the lung surface and the fistula site with chest wall soft tissue. We also used a pedicled serratus anterior muscle flap to fill the extrapleosteal space, specifically aiming to cover the fistula site. For the extrapleosteal space that could not be filled, we utilized air filling to promote lung re-expansion. Vancomycin was administered perioperatively, and the pyothorax cavity was irrigated with gentian-violet solution, which has been reported to have antimicrobial effects against MRSA [4]. However, it is important to note that Methylrosaniline chloride (also known as Gentian Violet, Crystal Violet, and marketed as Pyoctanin) faced regulatory changes. On December 28, 2021, the Ministry of Health, Labour and Welfare1, Japan issued a notification resulting in the general prohibition of its use due to risks of genetic toxicity and carcinogenicity. Furthermore, sales were discontinued by the end of the year 2022.

**Fig. 2:**
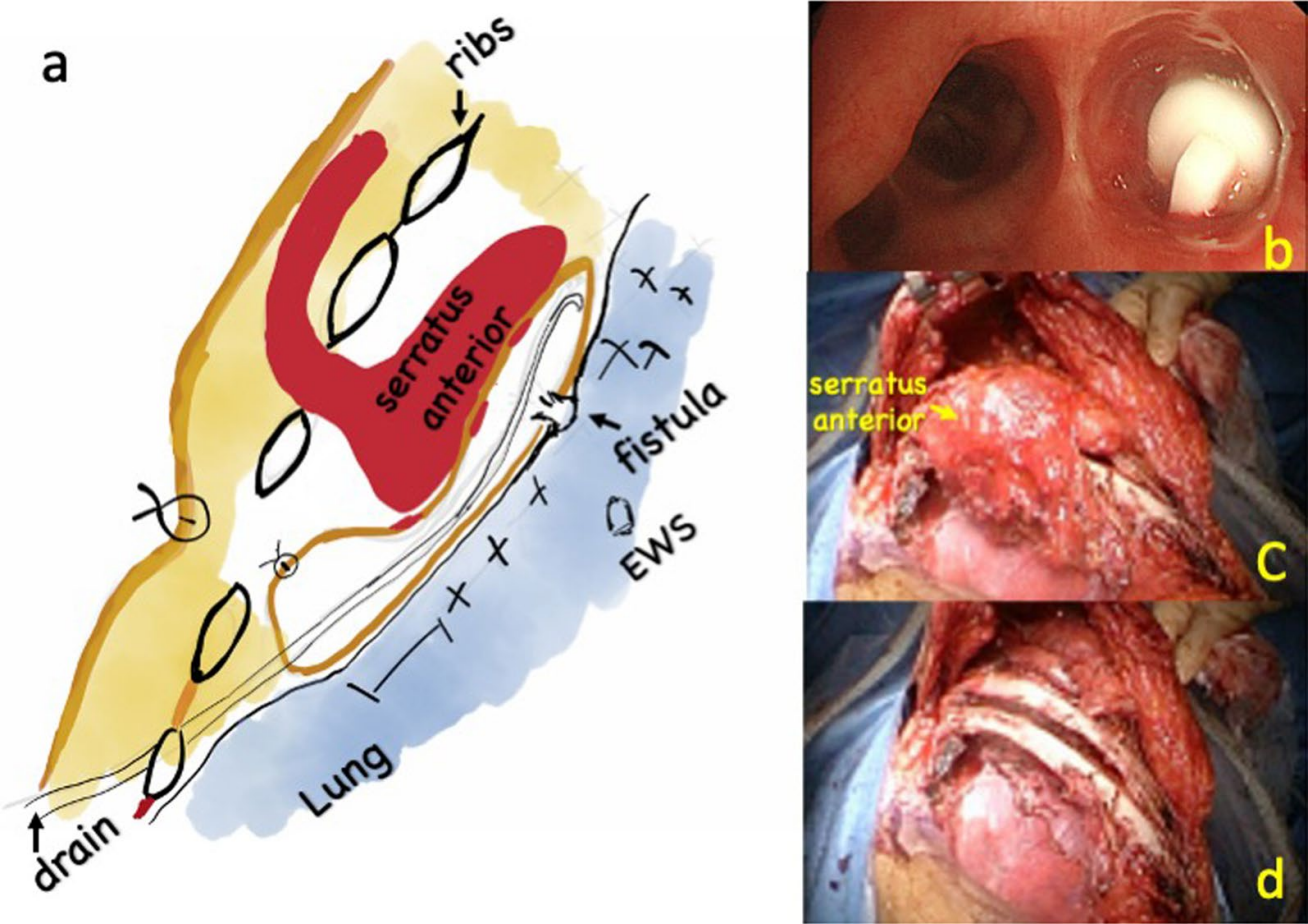
Illustration showing the procedure of window closing. **a** EWS was placed in the right B6 (**b**). Serratus anterior pedicle flap was created (**c**), and introduced into the extraperiosteal space through the intercostal gap (**d**)

**Fig. 3:**
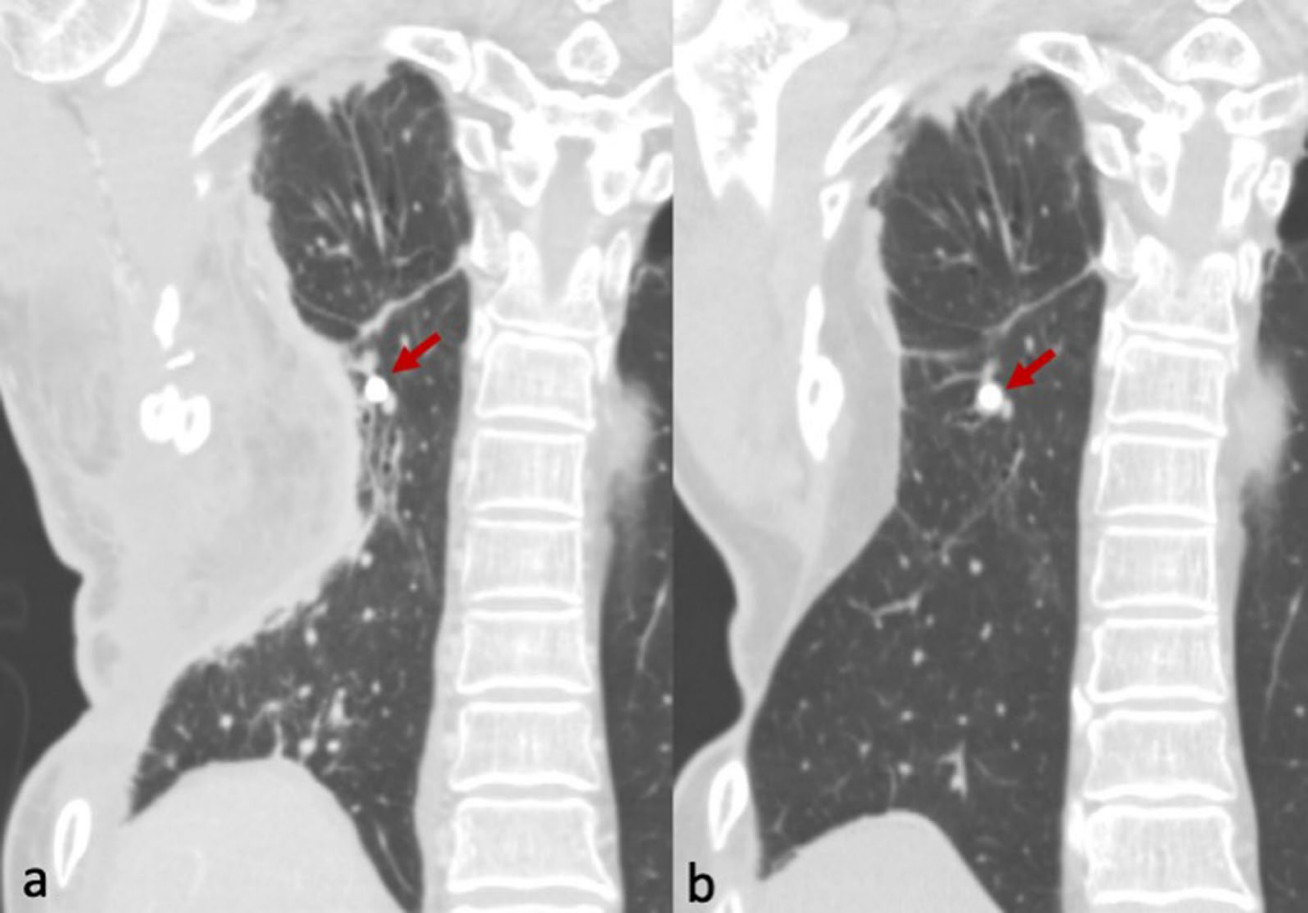
CT images: Immediate post-window closing surgery (**a**) and six years follow-up (**b**). The lung demonstrates good expansion, and the EWS remains preserved without any complications (arrows)

As a result, the closure surgery was successful, the pyothorax was cured, and long-term CT scans showed sustained lung expansion (Fig. 2). Although the EWS filled in the right B6a + b was naturally expectorated shortly after filling, the EWS filled in the right B6 remained in place for an extended period (Fig. 3a, b).

Although we also considered to perform a simple closure using a serratus anterior muscle flap without pleural stripping, preoperative evaluation revealed the presence of at least two fistulae. Therefore, we determined that suturing or muscle filling alone would be insufficient and opted for the coverage with soft tissues of the chest wall such as intercostal muscles and thickened parietal pleura. Moreover, EWS has been reported to be used for a prolonged period without complications [5], and in this case, we have been monitoring it without removal. At the 6-year follow-up, there have been no signs of infection recurrence or other complications. While there have been reports of combining EWS with Negative Pressure Wound Therapy (NPWT) to promote the reduction of pyothorax cavity before performing closure surgery [6], the duration of the window was prolonged in this case, and the visceral pleura outside the fistula site was covered with epithelialized tissue. Therefore, it was deemed unlikely to achieve granulation tissue growth even with NPWT, and we chose to proceed with the closure using the air plombage technique.

## Conclusion

We successfully achieved the closure of window thoracostomy in a patient with MRSA pyothorax with bronchopleural fistula. By utilizing EWS bronchial occlusion and muscle filling in the extraperiosteal pace, we effectively controlled lung fistula and infection. In addition, our modified air plombage technique, combined with other devices and methods, allowed for successful treatment, reducing thoracic deformities, and promoting lung re-expansion.

## Data Availability

Not applicable.

## Abbreviations

MRSA: Methicillin-resistant *Staphylococcus aureus*
EWS: Endobronchial Watanabe Spigot
CT: Computed tomography
NPWT: Negative pressure wound therapy
